# Polymer-Bioactive Glass Composite Filaments for 3D Scaffold Manufacturing by Fused Deposition Modeling: Fabrication and Characterization

**DOI:** 10.3389/fbioe.2020.00552

**Published:** 2020-06-24

**Authors:** Thomas Distler, Niklas Fournier, Alina Grünewald, Christian Polley, Hermann Seitz, Rainer Detsch, Aldo R. Boccaccini

**Affiliations:** ^1^Department of Materials Science and Engineering, Institute of Biomaterials, Friedrich-Alexander-University Erlangen-Nuremberg, Erlangen, Germany; ^2^Chair of Microfluidics, Faculty of Mechanical Engineering and Marine Technology, University of Rostock, Rostock, Germany

**Keywords:** 3D printing, fused deposition modeling, 3D printing filaments, bioactive glass, polymer ceramic composites, bone tissue engineering

## Abstract

Critical size bone defects are regularly treated by auto- and allograft transplantation. However, such treatments require to harvest bone from patient donor sites, with often limited tissue availability or risk of donor site morbidity. Not requiring bone donation, three-dimensionally (3D) printed implants and biomaterial-based tissue engineering (TE) strategies promise to be the next generation therapies for bone regeneration. We present here polylactic acid (PLA)-bioactive glass (BG) composite scaffolds manufactured by fused deposition modeling (FDM), involving the fabrication of PLA-BG composite filaments which are used to 3D print controlled open-porous and osteoinductive scaffolds. We demonstrated the printability of PLA-BG filaments as well as the bioactivity and cytocompatibility of PLA-BG scaffolds using pre-osteoblast MC3T3E1 cells. Gene expression analyses indicated the beneficial impact of BG inclusions in FDM scaffolds regarding osteoinduction, as BG inclusions lead to increased osteogenic differentiation of human adipose-derived stem cells in comparison to pristine PLA. Our findings confirm that FDM is a convenient additive manufacturing technology to develop PLA-BG composite scaffolds suitable for bone tissue engineering.

## Introduction

Bone is known for its self-healing abilities (Bose et al., 2013). The healing of bone fractures is a remarkable repairing process, resulting in the complete reconstruction of the tissue achieving its original form and functionality (Kumar and Narayan, 2014). Bone healing is a well-orchestrated process and for most minor fractures a mechanical fixation of the damaged bone region is sufficient for successful convalescence. However, if a defect reaches a critical size (~≥2.5 cm (Schemitsch, 2017; Nauth et al., 2018), depending on the surgical case), the endogenous regenerative capacity of bone tissue is insufficient for self-repair (Mothersill et al., 1991). Critical size bone defects caused by diseases such as osteogenesis imperfecta, osteoarthritis, osteomyelitis, osteoporosis, or conditions related to infection or induced by wear, still remain key challenges to be addressed in clinical practice (Porter et al., 2009; Nauth et al., 2018). Besides illnesses, trauma and tumors can lead to a critical size bone defect (Porter et al., 2009). The gold standard treatment involves autografts (bone taken from the patient's own body) and allografts (bone tissue taken from a donor) (Bose et al., 2013). Even if successful, challenges like the limited supply of autografts, transmission of diseases, rejection of grafts, donor site pain and morbidity, limitation in volume of donor tissue that can be safely harvested, and the possibility of harmful immune responses to allografts, drive surgeons and engineers to seek for alternative methods and materials to repair bone defects (Crane et al., 1995; Hill et al., 1999; Linero Palacios et al., 2002; Palmer et al., 2008; Garg et al., 2012). With the availability of novel manufacturing technologies like additive manufacturing (AM) (e.g., 3D-printing), new approaches to design and create engineered biomaterial alternatives to autografts and allografts have started to be developed (Bose et al., 2013; Ibrahim, 2017). Combining 3D printed scaffolds with cells, biotechnological platforms arise in which cells may proliferate, grow, and remodel to potentially develop 3D bone tissue analogs in a tissue engineering approach (Langer and Vacanti, 1993; Mantalaris et al., 2004; Salgado et al., 2004). Through AM and computer aided design (CAD), the fabrication of scaffolds with complex internal pores and shapes (architecture) as well as scaffolds catering to patient-specific needs are possible (Bose et al., 1999, 2003, 2013; Hutmacher et al., 2004). The AM of polymer-based scaffolds for bone engineering has been demonstrated utilizing various techniques (Hutmacher, 2000; Simon et al., 2006; Bose et al., 2013; Ibrahim, 2017; Tappa and Jammalamadaka, 2018). Among others, fused deposition modeling promises to be a solvent-free 3D printing approach with the potential to create patient-specific polymer-based biomaterial scaffolds (Hutmacher, 2000; Bose et al., 2013). FDM is based on the 3D printing of prior fabricated thermoplastic filaments which are subsequently processed in a second step using a hot extrusion nozzle to fabricate 3D structures without the use of a solvent (Hutmacher, 2000; Bose et al., 2013). Hutmacher (2000) demonstrated the 3D printing of polycaprolactone (PCL)-hydroxyapatite composites via FDM. Besides hydroxyapatite, bioactive glass is a well-known osteoinductive and osteoconductive material (Hench et al., 1971; Xynos et al., 2000; Hench, 2006). Combined with biopolymers, BG has been used to develop composite scaffolds for bone engineering (Hench, 2006; Chen et al., 2008; Gerhard and Boccaccini, 2010; Fu et al., 2011; Hench and Jones, 2015; Barbeck et al., 2017). Direct solvent-assisted printing has been demonstrated to successfully process polymer-BG composites (Russias et al., 2007; Bose et al., 2013; Murphy et al., 2016; Ibrahim, 2017). Exemplarily, Russias et al. (2007) showed solvent-based robocasting of PLA-BG and PCL-BG composites. Murphy et al. (2016) processed PCL-borate BG composites by mixing BG particles with PCL dissolved in chloroform to form a printable paste. Kolan et al. (2017) printed PCL-borate BG composites alternating with a Pluronic F127 support to produce 3D scaffolds through pressure-based extrusion. Barbeck et al. (2017) printed PLA/polyethyleneglycol (PEG)/calcium-phosphate-glass using PEG as a plasticizer to allow better rheological properties for direct extrusion. Eqtesadi et al. (2016b) robo-casted BG scaffolds followed by infiltration of PLA to improve the mechanical properties of the 3D printed BG scaffolds. One advantage of FDM over direct extrusion methods is the intermediate filament production step, allowing to achieve potentially storable filament materials for high throughput fabrication of reproducible scaffolds using FDM 3D printers. The FDM of PLA-BG has been demonstrated by Estrada et al. (2017), showing that the scaffolds were bioactive. However, the characterization of PLA-BG composite filaments for 3D printing, the reproducible fabrication of porous scaffolds and the assessment of the scaffold mechanical properties, cytocompatibility and osteoinductivity remain to be addressed to prove PLA-BG scaffold applicability for bone engineering. Among highly proliferative and available osteoblastic cell lines, murine pre-osteoblast cells (MC3T3E1) have been frequently used to study the cytocompatibility of biomaterials for bone engineering *in vitro* (López-Álvarez et al., 2013; Fu et al., 2017; Liu et al., 2018). Their main advantage is their potential for osteogenic differentiation in comparison to e.g., MG-63 cells which are arrested in pre-osteoblastic state (Czekanska et al., 2012). The potential of adipose-derived stem cells (ASC) for bone engineering has been recently highlighted (Vishnubalaji et al., 2012; Bhattacharya et al., 2016; Iaquinta et al., 2019; Storti et al., 2019) and ASC have been applied on biomaterial scaffolds as a potential critical size defect treatment strategy (Du et al., 2018). The high availability of ASC from body lipoaspirates (Yang et al., 2019) combined with the potential for osteogenic differentiation (Zhang et al., 2015) and defect reconstruction (Mesimäki et al., 2009; Yoshida et al., 2019; Zang et al., 2019) *in vivo* renders ASC excellent candidates to study the osteoinductive properties of biomaterials, with promising implications towards clinical translation (Barba et al., 2017). The aim of this study was to fabricate filaments for high throughput FDM of polymer-BG composite scaffolds with bioactive, cytocompatible, and osteoinductive properties. Composite filaments made from PLA and 45S5 BG were produced. The composite filaments were used for the FDM of porous scaffolds with bioactive and osteoinductive properties. 3D printed scaffolds were studied regarding their physicochemical properties as well as cytocompatibility and osteoinductivity using MC 3T3-E1 cells and human ASC.

## Materials and Methods

### Fabrication of PLA-Bioactive Glass (BG) Filaments

Composite filaments were produced using PLA as the bulk matrix material and BG as a filler. 45S5 BG (composition: 45 wt% SiO_2_−24.5 wt% CaO−24.5 wt% Na_2_O−6 wt% P_2_O_5_, d50: (4.0 ± 1.0) μm, d95: ≤ 20 μm, Schott Vitryxx^®^, Schott AG, Germany) was used. A PLA powder was selected (PLA RXP 7503, Resinex GmbH, Germany). To prevent BG particle agglomeration, the glass was sieved through a 80 μm mesh (Mini-Sieve Micro Sieve Set, SP Scienceware—Bel-Art Products, USA) and treated with an anti-static ionizer (STABLO-AP, Shimadzu Cooperation, Japan) prior to mixing. PLA (100 g) was mixed with 0, 1, 2.5, 5, and 10% (wt) of 45S5 BG by equal distribution in five 50 ml cell culture tubes (SARSTEDT AG & Co. KG, Germany) filling ~25 ml of the tubes and subsequent rotationally mixing (Intelly-Mixer, ELMI, Latvia) at 60 rpm for 30 min. The powder was poured into the hopper of a desktop filament extruder (NEXT 2.0, 3Devo B.V., Netherlands). The material was fed in small portions of 10–20 g to reduce the time the material would spend in the hopper to prevent heat associated material agglomeration. The extrusion screw was always covered with layers of PLA-45S5 BG to ensure constant material intake. Cooling fans of the extruder were turned on as soon as the filament diameter reached a value ≥ 1 mm. After reaching a stable target diameter of 2.85 mm, the filament was collected on a spool. Filaments with a tolerance of ± 0.15 mm were considered suitable for final scaffold printing. The data produced by the optical sensor was monitored by a desktop computer connected to the extruder during filament production. Between each filament production process, the extruder was purged using high molecular weight polyethylene (HMWPE, 3Devo B.V., Netherlands) cleaning polymer. PLA filaments with varying BG contents were created ranging from 0, 1, 2.5, 5 up to 10 wt%. The final heating parameters were 110, 155, 155, and 145°C for heaters 4 to 1, respectively, with heater 4 being the heater closest to the hopper, heater 1 being the heater closest to the extrusion nozzle. Screw speed was set to 5.6 rpm, the fan speed was set to 50% of the maximum possible fan speed of the extruder.

### Filament Characterization

#### Light Microscopy

Filament diameter, morphology and optical appearance were assessed using a Stemi 508 (Carl Zeiss, Jena) light microscope followed by Image processing via the ImageJ software package (Fiji, ImageJ 1.52i).

#### Scanning Electron Microscopy (SEM)

To assess BG particle distribution inside PLA-BG composite filaments, scanning electron microscopy (SEM) was performed (Auriga CrossBeam, Carl Zeiss microscopy GmbH, Germany). Fracture surfaces of PLA-BG filaments were prepared by immersion of the filaments in liquid nitrogen (LN2) at ~-180°C and subsequently breaking them manually prior to SEM imaging.

#### Tensile Testing

The mechanical tensile properties of PLA-1, 2.5, 5, 10% (wt) BG composite filaments were determined using a universal testing machine (Zugfestigkeitsprüfmaschine Model FRANK, Karl Frank GmbH, Mannheim, Germany). Filaments (*n* = 6) were mounted using a 1kN sample holder at 3.5 bar, with tensile tests being recorded using a 1kN load cell and a constant deformation speed of 10 mm.s^−1^, according to DIN53455.

### Printability Assessment

To determine the accuracy of 3D printing using the fabricated PLA-BG composite filaments, a printability assessment was performed. A resolution tree was 3D printed to evaluate printing resolution using the manufactured PLA-BG filaments. In the resolution tree test, strut distances between 1 mm and 200 μm were examined, with the strut width set to 0.4 mm and strut distances reducing in increments of 100 μm and 10 μm to determine the zone of strut merging. Resolution trees were examined via a light microscope and images were processed using ImageJ. The strut width of *n* = 6 struts was measured as well as the position at which two struts would merge for the first time. The strut distance before merging was considered the resolution limit. To evaluate printability regarding 3D cylindrical open-porous scaffolds, samples (*n* = 4) were 3D printed and the porosity of the top and the side of the scaffold was assessed via ImageJ. Subsequently, the pore area (*n* = 6) was calculated and the deviation (d_ev_) from the theoretical pore size given by CAD model (750 μm) was determined using the following equation, as described by Tappa and Jammalamadaka (2018):

$$\begin{array}{l} d_{ev}=\frac{ARt-ARe}{ARt}*100\%\; \end{array}$$

where A_Rt_ is the theoretical pore area and A_Re_ is the experimental pore area measured from 3D printed scaffolds.

### Scaffold Fabrication Using PLA-BG Filaments

Cylindrical scaffolds (diameter = 10 mm, height = 12 mm) were designed with an interconnected porosity and pore diameter of 750 μm using computer aided design software solid edge (Siemens AG, Germany) and the browser-based CAD tool tinkercad (Autodesk Inc., USA). PLA-BG filaments with 0, 1, 2.5, 5, and 10% (wt) BG content were fed into a FDM 3D printer (Ultimaker S5 Premium, Ultimaker B.V., Netherlands) and scaffolds were produced. The detail printing parameters can be found in Supplementary Table 1. The printer was equipped with an extrusion nozzle of diameter D = 400 μm, and a tempered glass building plate. No features of the 3D CAD design were smaller than the resolution limit of the FDM-printer of 0.4 mm.

### Micro-CT (μCT) Imaging

To investigate the BG distribution and interconnectivity of porosity of 3D printed PLA-BG scaffolds, μCT analysis was performed. μCT tomograms of PLA-1%(wt) BG scaffolds were recorded on a Skyscan 1076 scanner (Bruker, Kontich, Belgium) applying a source voltage of 55 kV and a source current of 181 mA. To reduce beam hardening artifacts, a 0.5 mm aluminum filter was used. The scan resolution was set to 9 μm per voxel. For noise reduction, an average of 4 frames was recorded every 0.6 degree. The scans were reconstructed applying the cone beam algorithm in the NRecon software package (Bruker, Kontich, Belgium). High resolution 3D renderings were created using CTVox software (Bruker, Kontich, Belgium).

### Mechanical Characterization

To evaluate the mechanical properties of the 3D printed scaffolds, compression strength tests were performed using an universal testing system (Instron 3300 Floor Model, Instron^®^ GmbH, Germany). The tests were carried out with a speed of 1.3 mm.min^−1^ in accordance to ASTM D695 (ASTM D695-15, 2015). The starting distance was set close to the height of the scaffolds and the total compression displacement was set to 3 mm. Scaffold surface area was measured prior to the mechanical assessment. Images of the scaffolds (*n* = 3) for each group were taken using a light microscope (ZEISS Stemi 508, Zeiss AG, Germany). The area of each sample was calculated in ImageJ software using the polygon selection tool.

### Bioactivity Study

For the bioactivity assessment of the PLA-BG scaffolds, simulated body fluid (SBF) was produced according to Kokubo and Takadama (2006) and as stated in ISO 23317 (ISO 23317:2014(E)H, 2014). A set of 3D printed PLA-BG squares of 6 × 6 × 0.4 mm^3^ (*n* = 6) was fabricated per group. The required amount of 9.6 ml of SBF was calculated using the formula stated by Kokubo and Takadama (2006). The equation describes the volume of SBF needed as:

$$\begin{array}{l} Vs=\frac{Sa}{10\;} \end{array}$$

where V_s_ is the volume of SBF in ml and S_a_ is the apparent surface area of the specimen in mm^2^. The samples were placed in SBF and put in a shaking incubator (Heidolph Unimax 1010, Heidolph Instruments GmbH & CO. KG, Germany) at 37°C and 90 rpm. SBF was changed every 2 days. Sets of samples (*n* = 3) per group were removed after 14 and 28 days of incubation in SBF. Samples were washed with ultrapure water and dried under a fume hood at 22°C (room temperature, RT). Before and after the SBF incubation, light microscopy images were recorded. After drying, the samples were characterized using Fourier transformed infrared spectroscopy (FTIR), X-ray diffraction (XRD), and energy dispersed x-ray (EDX) analyses. The chemical composition of pristine and SBF incubated PLA-BG samples was characterized by FTIR (IRAffinity-1S, Shimadzu Europa GmbH). Absorbance spectra of PLA-BG were recorded after 0, 7 and 14 days of incubation in SBF. Samples were also tested with XRD (MiniFlex 600, Rigaku Corporation, Europe) to characterize the crystallinity of the surface layer after SBF incubation. Angles 2Θ of 20–80° were investigated, with 0.02° per step and a speed of 2° per minute. EDX was used to evaluate the composition of the surface of SBF incubated samples using an EDX system (X-Max^N^, Oxford Instruments) fitted in a scanning electron microscope (Auriga Crossbeam, Carl Zeiss Microscopy GmbH, Germany). EDX spectra were recorded on non-sputtered samples at a working distance of 6 mm and an accelerating voltage of 10 keV to determine elemental surface composition. Map and point scans were performed at a dwell time of 10 μs.

### Cell Culture Studies

#### Cell Culture

Mouse calvaria pre-osteoblast MC3T3E1 cells (Sigma Aldrich, Germany) were used to assess cytocompatibility of the 3D printed PLA-BG scaffolds. The cells were cultured in alpha-modified minimum essential medium (α-MEM) (Gibco^®^, Life Technologies™, Germany) containing 10% (v/v) fetal bovine serum (FBS, Sigma-Aldrich), 1% (v/v) penicillin/streptomycin (Sigma-Aldrich, Germany) and 1% (v/v) L-Glutamine (Thermo Fisher Scientific Inc., USA) media supplements. Cells were passaged in T75 cell culture flasks (Sarstedt, Germany) at 37°C and in a humidified atmosphere of 95% air and 5% CO_2_ in an incubator (Galaxy^®^ 170 R, Eppendorf AG, Germany). For cell detachment, Trypsin/EDTA (Sigma Aldrich, Germany) was used with cell counting performed using the trypan blue exclusion method using Neubauer chambers (Paul Marienfeld GmbH & Co.KG). To evaluate cell differentiation and gene expression on the composite materials, human adipose-derived stem cells were used (Lonza, CH). The cells were passaged in phenol-red free Dulbecco's modified eagle medium (DMEM) containing 10% (v/v) fetal calf serum (FCS, Corning, USA) and 1% (v/v) penicillin/streptomycin (Thermo Fisher, USA). Cells were harvested and counted using Trypsin/EDTA (Thermo Fisher, USA) and the trypan blue exclusion method. Human ASC at passage 4 (p4) were seeded on 3D printed PLA-1% BG scaffolds (150,000 cells/scaffolds) and cultured for 35 days in maintenance (-OS) and osteogenic (+OS) differentiation medium at 37°C in a humidified atmosphere of 95% air and 5% CO_2_ in an incubator. Human ASC seeded on 3D printed PLA scaffolds without BG served as material controls. Osteogenic (+OS) medium consisted of phenol red DMEM containing 10% FCS, 1% penicillin/streptomycin, 50 μg.ml^−1^ ascorbic acid, 10 mM beta-glycerolphosphate, and 10 mM dexamethasone (all Sigma Aldrich). Non-osteogenic (-OS) medium contained phenol red DMEM, 10% FCS, and 1% penicillin/streptomycin.

#### *In vitro* Cytocompatibility

For the *in vitro* cytocompatibility assessment, two different structures of PLA-BG composites were 3D printed. Cylindrical scaffolds with three layers, a total height of 2.25 mm and a diameter of 10 mm with interconnected porosity as well as cell culture disks with a height of 4 mm and a diameter of 13 mm were produced. The disk surface was printed with a parallel line infill pattern. As a result, the disk featured an orientated topography to test the ability of directional guidance in cell growth. 3D PLA-BG scaffolds and cell-culture disk containing 0, 1, 2.5, 5, and 10% (wt) BG (*n* = 6) were printed and disinfected using UV light exposure. Wettability of the materials as well as pH development of cell culture medium (5 ml, *n* = 3 scaffolds) in contact with the scaffolds was recorded prior to cell culture. The scaffolds and cell culture disks were placed in 24-well-plates (Sarstedt, Germany) and MC3T3E1 cells were seeded with a concentration of 100,000 cells.ml^−1^ (Brooks et al., 2016). All samples were cultured for 24 h to assess initial cell attachment and *in vitro* cytocompatibility. Tissue culture polystyrene (PS) substrates served as additional controls to the cell-culture disks.

#### Cell Viability

To assess cell viability, a water-soluble tetrazolium salt (WST-8) assay was performed to indirectly determine the viability of cells on the different substrates by conversion of a water-soluble tetrazolium salt through cellular metabolism into an insoluble formazan. After 24 h, the medium was removed from the cells and the cells on scaffolds (*n* = 6) were incubated with cell culture medium containing 1% (v/v) WST solution (Cell Counting Kit - 8, Sigma Aldrich, Germany) for 3 h according to manufacturer's instructions. An equally incubated WST-8 master stock solution served as control. After incubation, 100 μl aliquots (technical duplicates) were transferred into a 96-well-plate (Sarstedt, Germany) and the absorbance at 450 nm was recorded using a plate reader (type Phomo, Anthos Mikrosysteme GmbH, Krefeld, Germany).

#### LIVE/DEAD Staining

To determine the cellular viability on the 3D printed disk, a live/dead staining assay was performed. Viable cells were stained by calcein acetoxymethyl ester (Calcein AM), while apoptotic and necrotic cells were stained by propidium iodide (PI) (both Invitrogen™, Molecular probes by Life technologies™, USA), corresponding to live and dead cells, respectively. The samples were washed with phosphate buffered saline (DPBS, Thermo Fisher, USA) and incubated with 1 ml of DPBS stock solution containing 4 μl.ml^−1^ Calcein AM and 5 μl.ml^−1^ PI for 45 min. After incubation, the samples were washed with DPBS and fixed using 500 μl of fixing solution containing 0.1 M PIPES (Piperazine-N,N′-bis(2-ethanesulfonic acid), Merck, Germany), 1 mM EGTA (Ethylene glycol tetraacetic acid, Merck, Germany), 4% (w/v) polyethyleneglycol, and 3.7% (w/v) paraformaldehyde (all Sigma Aldrich, Germany), dissolved in HBSS. After 5 min of fixing, the samples were washed with DPBS and examined using a fluorescence microscope (FM) (Scope.A1, Carl Zeiss, Germany). Cell nuclei of fixed cells on 3D printed scaffolds were stained using Hank's buffered salt solution (HBSS) containing 1 μl.ml^−1^ DAPI (4′,6-diamidino-2-phenylindole, Invitrogen™, USA) for 5 min.

### Gene Expression Analysis

For cDNA synthesis, total RNA was extracted from human ASC cultured on PLA and PLA-1% BG scaffolds (*n* = 6) using a RNeasy mini kit (Qiagen, Germany) according to the manufacturer's instructions. Cells were detached from the scaffolds using Trypsin/EDTA (ThermoFisher, USA), pelleted by centrifugation, and lysed using RLT buffer (RNeasy mini kit). RNA concentration and quality were quantified using a NanoDrop™ One (ThermoFisher Scientific, USA) spectrophotometer. cDNA was reverse-transcribed from 150 μg RNA using iScript Advanced Reverse Transcription Supermix (Bio-Rad, Germany) according to the manufacturer's instructions. Real-time quantitative PCR (RT-qPCR) was performed on a CFX96 thermocycler (Bio-Rad, Germany) to measure levels of gene expression using SsoAdvanced Universal SYBR Green Supermix (Bio-Rad, Germany) on six replicate samples in technical duplicates with human primePCR validated specific primers (Supplementary Table 2). Relative gene expression was quantified by the 2^−ΔΔ*Cq*^ method and normalized using YWHAZ, HPRT1 and GAPDH multiple housekeeping genes. Relative gene expression of alkaline phosphatase (ALPL), Runt-related transcription factor 2 (RUNX2), collagen type I (COL1), osteocalcein (BGLAP) and vascular endothelial growth factor A (VEGF) was analyzed. Data analysis was conducted using the CFX Maestro software package (Bio-Rad, Germany). Light microscopy images were taken during and after 35 days of incubation to assess cellular growth on the scaffolds.

### Statistical Analysis

All experiments were conducted using at least three replicate scaffolds per group. Statistical analyses were performed using one-way analysis of variances (ANOVA) with *post-hoc* Tukey test for multiple comparison of means between normally distributed groups and Welch's *t*-test for pairwise comparison between two groups using Origin 2019 software (OriginLab Corporation, Northhampton, USA). Data are expressed as mean ± SD except gene expression analysis where data are expressed as mean ± s.e.m. Number of samples per group were *n* = 6 (filament diameter), *n* = 6 (filament tensile testing), *n* = 6 (filament printability), *n* ≥ 4 (scaffold characterization), *n* = 6 (scaffold mechanics), *n* = 6 (bioactivity assessment), *n* = 3 (pH), *n* = 6 (*in vitro* characterization), *n* = 6 (gene expression analyses). Differences were considered significant with ^*^p < 0.05, ^**^p < 0.01, ^***^p < 0.001, ^****^p < 0.0001.

## Results

### Filament Production

**Figure 1:**
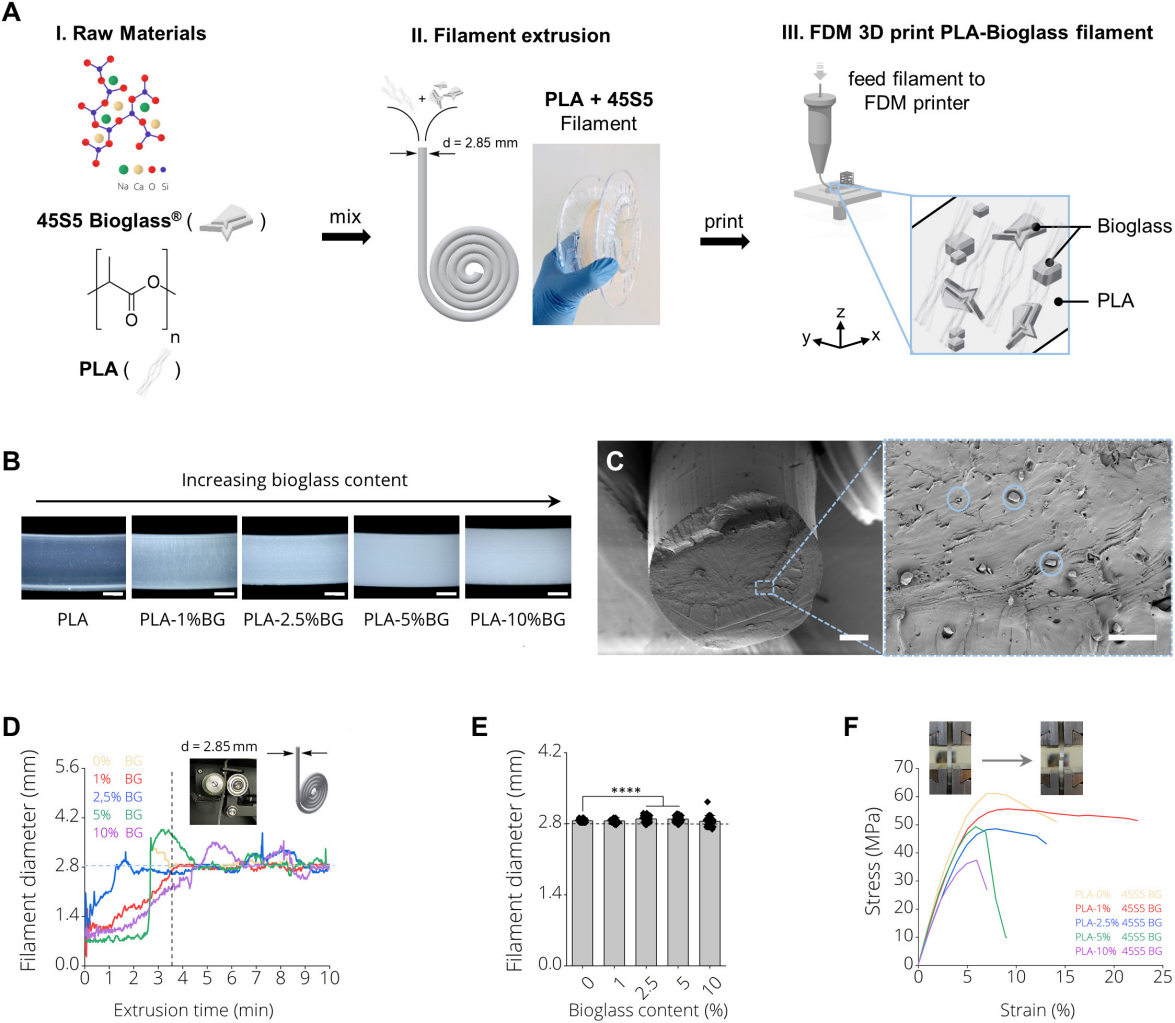
PLA-45S5 BG filaments for fused deposition modeling. **(A)** Schematic diagram depicting the process pipeline to fabricate PLA-BG FDM printed scaffolds with (i) mixing of the raw materials PLA and 45S5 BG, (ii) filament production using a polymer extruder with an image of the final extruded and spooled 45S5 BG containing PLA filaments and (iii) FDM 3D printing by feeding the prior produced filaments into a FDM printer. **(B)** Light microscopy images of the final BG-laden PLA filaments of 0, 1, 2.5, 5, and 10% (wt) 45S5 BG content. An increase in turbidity with increasing BG content is observed. Scale bars: 500 μm. **(C)** SEM Micrograph of the filament cross section with 45S5 BG (d_50_ = 4 ± 1 μm) particles homogeneously distributed inside the bulk PLA matrix. Scale bars: 500 μm (left), 15 μm (right). **(D)** Filament diameter monitored during filament extrusion illustrating the yield of filament as a function of the time required until a near constant filament diameter of d = 2.85 mm was extruded. It is visible that with increasing BG content, the time required to extrude filaments of a constant diameter increases. **(E)** Diameter of final filaments utilized for FDM printing (mean ± SD, *n* = 6; ^****^p < 0.0001 significant difference of mean diameter compared to pure PLA filaments determined by one-way ANOVA). **(F)** Tensile testing of the resulting PLA-45S5 BG filaments. Representative stress strain curves depicting the characteristic stress strain behavior of the resulting filaments with a decrease in ultimate tensile strength and elongation at break with increasing BG content.

Figure 1A depicts the production process of 3D printing filaments made by (I) mixing PLA and 45S5 BG particles, (II) filament extrusion using a desktop filament extruder and (III) 3D printing of the produced filaments using FDM. Figure 1A, II shows a final BG containing filament on a carrier spool ready for subsequent FDM printing. It was possible to produce filaments of PLA with increasing BG content of 1, 2.5, 5, and 10% (wt) BG (Supplementary Figure 2). We observed an increase of turbidity in the filaments with increasing amount of BG (Figure 1B). Scanning electron microscopy micrographs indicated a homogenous distribution of BG particles (d_50_ = 4 ± 1 μm) inside PLA-BG filaments (Figure 1C). Melts of PLA-BG mixtures were extruded from the extruder and monitored live over time to assess the time point after which the goal filament diameter of d = 2.85 mm was achieved for each PLA-BG composition. The continuous monitoring allowed to assess the deviation in filament diameter from the filament extruder over time as a measure of the process stability. Figure 1D shows the filament diameter of the differently BG-laden PLA filaments over extrusion time. We found that with increasing BG content [>2.5% (wt) BG, Figure 1D, blue, green, purple graph], the deviation of filament diameter around the diameter aimed at (d = 2.85 mm) increased significantly in comparison to PLA-0% BG and PLA-1% BG filaments (Figure 1E). It was possible to produce filaments of all BG filler contents around the aimed filament diameter suitable for FDM using the herein utilized 3D printer. During filament extrusion, filament adhesion on the puller wheel (Supplementary Figure 1) of the NEXT 2.0 filament extruder was observed. A tool to be attached on NEXT 2.0 filament maker models to avoid this adhesion is provided (Supplementary Figure 1). (The file is available in the Supplementary Materials to this article as an open source ready-to-print ^*^.stl file). The mechanical properties of the BG containing PLA filaments were assessed via tensile testing. It was found that with BG contents exceeding 1% (wt), tensile strength and toughness decreased (Figure 1F), which is most likely associated to insufficient BG bonding to the PLA matrix, observed in SEM cross sections (Figure 1C).

### Printability of PLA-BG Filaments

**Figure 2:**
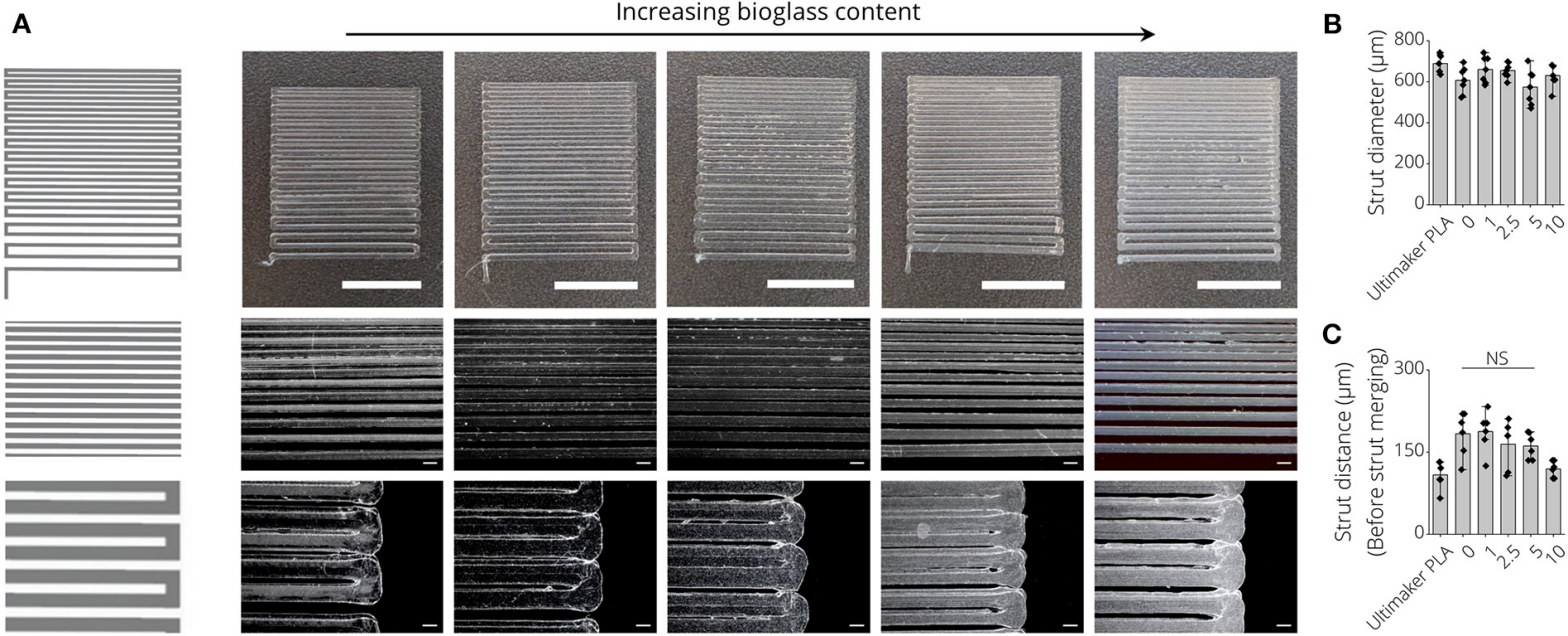
Printability assessment of PLA-BG filaments. **(A)** CAD model (left) and light microscopy images (right) of the FDM printed BG-laden PLA filaments into meandering structures. Scale bars: 2000 μm (top row), 500 μm (mid row), 200 μm (bottom row). The light microscopy images indicate strut merging and increase in turbidity with increasing BG content. **(B)** Strut diameter achieved of 3D printed PLA-BG filaments with increasing BG content of 0, 1, 2.5, 5, and 10% (wt) BG in PLA, using a diameter = 400 μm FDM nozzle (*n* = 7, mean ± SD). **(C)** The distance between struts before strut merging occurred processing PLA-BG filaments. Strut distance before merging indicated the highest resolution possible between two struts for each material (*n* = 6, mean ± SD). NS indicates no significant difference comparing the different groups with *p* < 0.05 by one-way ANOVA test.

To assess the printability of PLA, PLA-1% BG, PLA-2.5% BG, PLA-5% BG, and PLA-10% BG filaments via FDM, resolution trees were fabricated from CAD models using the different filaments (Figure 2A). In light microscopy images, an increasing turbidity of the printed structures, indicative of the higher loading of BG particles, was observed (Figure 2A). PLA filaments provided by the 3D printer supplier (Ultimaker) served as commercial PLA printing control. We found that the strut diameter of 3D printed resolution trees of all processed filaments was around 625 ± 68 μm without statistically significant differences between the groups (Figure 2B). Assessing the most narrow distance between struts that could be printed before strut merging occurred, the fabricated filaments in this study showed a maximal printing resolution of 163 ± 27 μm, with no significant difference between PLA-BG filaments of pristine PLA, 1, 2.5, and 5% BG loading (Figure 2C). Printing the commercially available PLA reference filament allowed a resolution of 108 ± 25 μm strut distance.

### Scaffold Fabrication From PLA-BG Filaments

**Figure 3:**
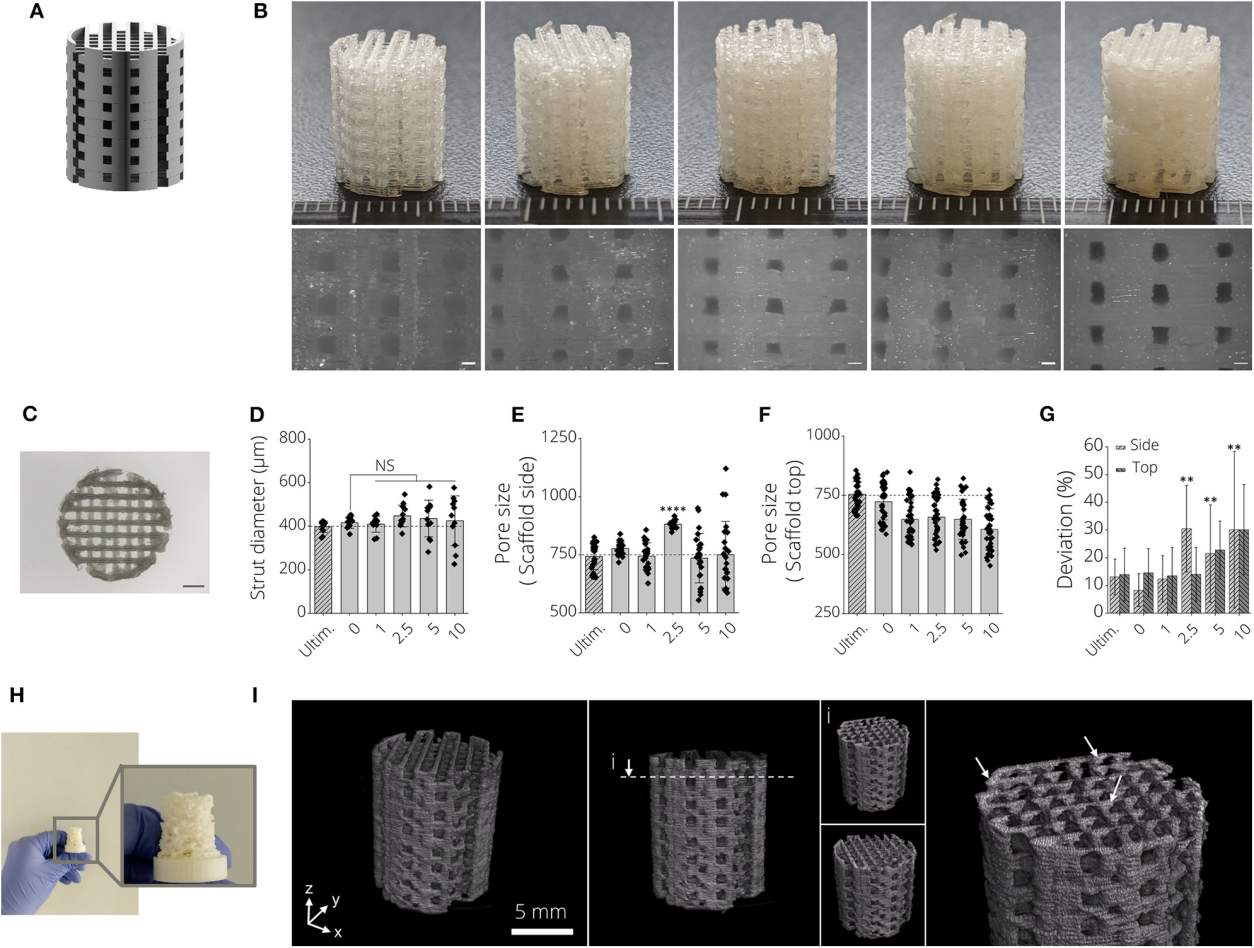
3D printed PLA-45S5 BG scaffolds. **(A)** CAD render of the aimed design for PLA-BG scaffolds with a pore size of width = 750 μm. **(B)** FDM 3D printed PLA-BG scaffolds from prior produced filaments. From left to right: PLA-0% (wt) BG, PLA-1%(wt) BG, PLA-2.5% (wt) BG, PLA-5% (wt) BG, and PLA-10% (wt) BG 45S5. Light microscopy images display the scaffold sides (bottom). Scale bars: 500 μm. **(C)** Top view light microscopy image of a PLA-BG scaffold. Scale bar: 2 mm. **(D–G)** Printability assessment and porosity analysis of PLA-BG scaffolds compared to Ultimaker PLA reference filaments depicting **(D)** strut diameter, **(E)** porosity at the side and top **(F)** of the scaffolds, as well as **(G)** the deviation of pore area from the theoretical pore area calculated from the CAD model as a measure of printing accuracy. ^**^p < 0.01, ^****^p < 0.0001 indicate statistical significant difference of means in comparison to 3D printed 0% BG PLA by one-way ANOVA or Welch's *t*-test in pairwise comparisons of scaffold side pore diameter. **(H)** Print of a complex shaped CAD model based on an upscaled MRI scan of a mouse femur. **(I)** μCT image of 3D printed PLA-1% BG scaffold showing the interconnected porosity and indicating even distribution of BG in the scaffolds (white arrows).

Open-porous three-dimensional structures were designed to produce biomaterial scaffolds from PLA-BG filaments (Figure 3A). It was possible to produce open porous scaffolds of height h = 12 mm, diameter d = 10 mm using all fabricated filaments in this study (Figure 3B). Light microscopy images indicated deviations from ideally rectangular pore geometries (Figure 3B, left, pristine PLA) when printing BG containing PLA filaments (Figure 3B). Figure 3C shows a top view light microscopy image of a 3D printed PLA-BG scaffold. The increase in turbidity with increasing BG content is visible, as well as material lumps deposited and defects introduced when fabricating higher BG content PLA-BG scaffolds from PLA-5% BG and PLA-10% BG filaments (Supplementary Figure 3). The strut diameter of the printed scaffolds varied negligibly from the theoretical strut diameter of 400 μm given by the 3D printers extrusion nozzle (Figure 3D). However, with increasing BG content, the variation in strut diameter increased, indicated by increasing standard deviation (SD) (Figure 3D). The mean strut diameter did not significantly change in comparison to 0% BG PLA scaffolds. Comparing to the CAD designed pore diameter (750 μm), the pore size of the top of the scaffolds prepared from PLA-BG did not significantly change except for PLA-2.5% BG scaffolds (Figure 3E). It was observed that with 5 and 10% BG containing PLA filaments, the SD of pore size increased, however not significantly altering the mean pore size. Regarding pore size assessed from the scaffold top, a reduction in pore size was observed with increasing BG content (Figure 3F). Figure 3G depicts the deviation of the pore size area in comparison to the theoretical pore size area designed in CAD (0.5625 mm^2^). With increasing BG content in PLA-BG filaments, an increase in the deviation from the theoretical pore size was observed (Figure 3G), with significant deviation from the theoretical pore area for 2.5, 5, and 10% (wt) BG containing PLA-BG filaments (^**^p < 0.01). It was possible to predict and tailor the pore size based on the CAD model for PLA-BG filaments with low BG content (PLA-1% BG). In an attempt to assess the capacity to print more complex geometries, structures like e.g., an upscaled μCT derived model from a mouse femur, was successfully printed (Figure 3H) from PLA-1% BG filaments. μCT images derived from reconstructed tomograms of PLA-1% BG scaffolds confirmed the interconnected porosity of the PLA-BG scaffolds (Figure 3I, Supplementary Video 1). White arrows depicting areas of higher x-ray absorbance in the images indicate the presence and homogeneous distribution of bioactive glass particles in the 3D printed scaffolds due to higher x-ray absorbance in comparison to bulk PLA (Figure 3I, Supplementary Video 2).

### Mechanical Properties of PLA-BG Scaffolds

**Figure 4:**
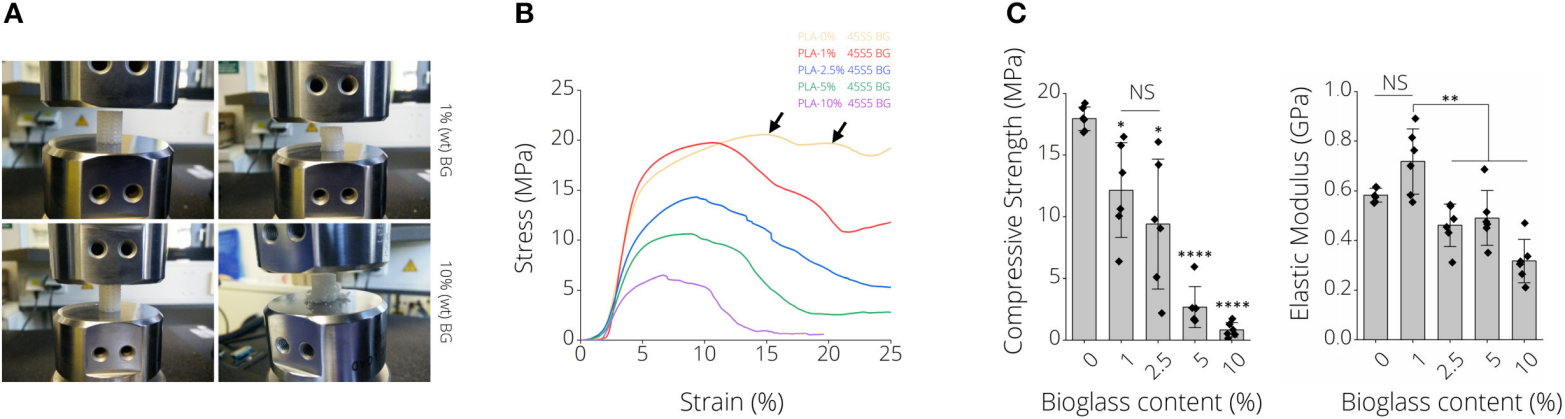
Mechanical properties of 3D printed PLA-45S5 BG scaffolds. **(A)** Macroscopic images illustrating different failure modes of 1 and 10% (wt) BG loaded PLA-BG scaffolds indicating buckling and brittle failure, respectively. **(B)** Qualitative stress strain diagram from compression tests of PLA-0, 1, 2.5, 5, and 10%-BG laden 3D printed scaffolds. A loss in scaffold toughness with increasing BG content is visible. The consecutive failure of different layers inside the scaffolds during compression testing is indicated by stress hills (black arrow) inside the diagram after initial failure of a scaffold layer. **(C)** Compression strength and modulus of elasticity of 3D printed PLA-BG scaffolds with increasing BG content (*n* = 6). Data is shown as mean ± SD. ^*^p < 0.05, ^**^p < 0.01, and ^***^p < 0.001 indicate statistical significant differences of means in comparison to 3D printed pristine PLA scaffolds by one-way ANOVA or Welch's *t*-test pairwise comparisons.

Figure 4A depicts representative images of scaffolds after compression tests, namely for PLA-1% BG and PLA-10% BG scaffolds. Differences in failure behavior from buckling (PLA-1% BG, Supplementary Video 3) to brittle fracture (PLA-10% BG) were observed (Figure 4A). Stress-strain diagrams show the decrease of work-of-fracture with increasing BG content in correspondence to those observations (Figure 4B). A multiple-stage failure process (black arrows) with regions of decreasing and increasing stress is visible for PLA-0% BG and PLA-1% BG scaffolds (Figure 4B), related to buckling and incremental failure of single struts observed during testing. For example, the compressive strength of PLA-BG scaffolds decreased from 18 ± 10 MPa (PLA) to 12 ± 4 MPa (^*^p < 0.05; PLA-1% BG) and 3 ± 2 MPa (^****^p < 0.0001; PLA-5% BG) with increasing BG content. We found a significant decrease in stiffness of BG-laden PLA scaffolds exceeding 1% (wt) BG (Figure 4C), with no significant difference in elastic modulus between pristine PLA (0% BG) and 1% (wt) PLA-BG scaffolds. A summary of the values of the 3D printed PLA-BG scaffolds can be found in Table 1. The elastic properties of PLA scaffolds loaded with 0–2.5% (wt) BG showed mechanical properties similar to the range of cancellous bone of human proximal tibias (Hvid et al., 1983; Rho et al., 1993).

**Table 1:** Mechanical properties of PLA-BG scaffolds.

**Sample**	**Elastic modulus (GPa)**	**Compressive strength (MPa)**
0 BG	0.6	18 ± 10
1 BG	0.7 ± 0.1	12 ± 4
2.5 BG	0.5 ± 0.1	9 ± 5
5 BG	0.5 ± 0.1	3 ± 2
10 BG	0.3 ± 0.1	1 ± 1
Cortical Bone	18 GPa−30 (Rho et al., 1993; Mohamed and Shamaz, 2014)	100–203 (Mohamed and Shamaz, 2014)
Trabecular Bone	0.5–1.5 (Hvid et al., 1983); 13–20 (Ashman and Rho, 1988; Oftadeh et al., 2014)	2–12 (Hvid et al., 1983; Røhl et al., 1991; Mohamed and Shamaz, 2014)

### Bioactivity of FDM Printed PLA-BG Filaments

**Figure 5:**
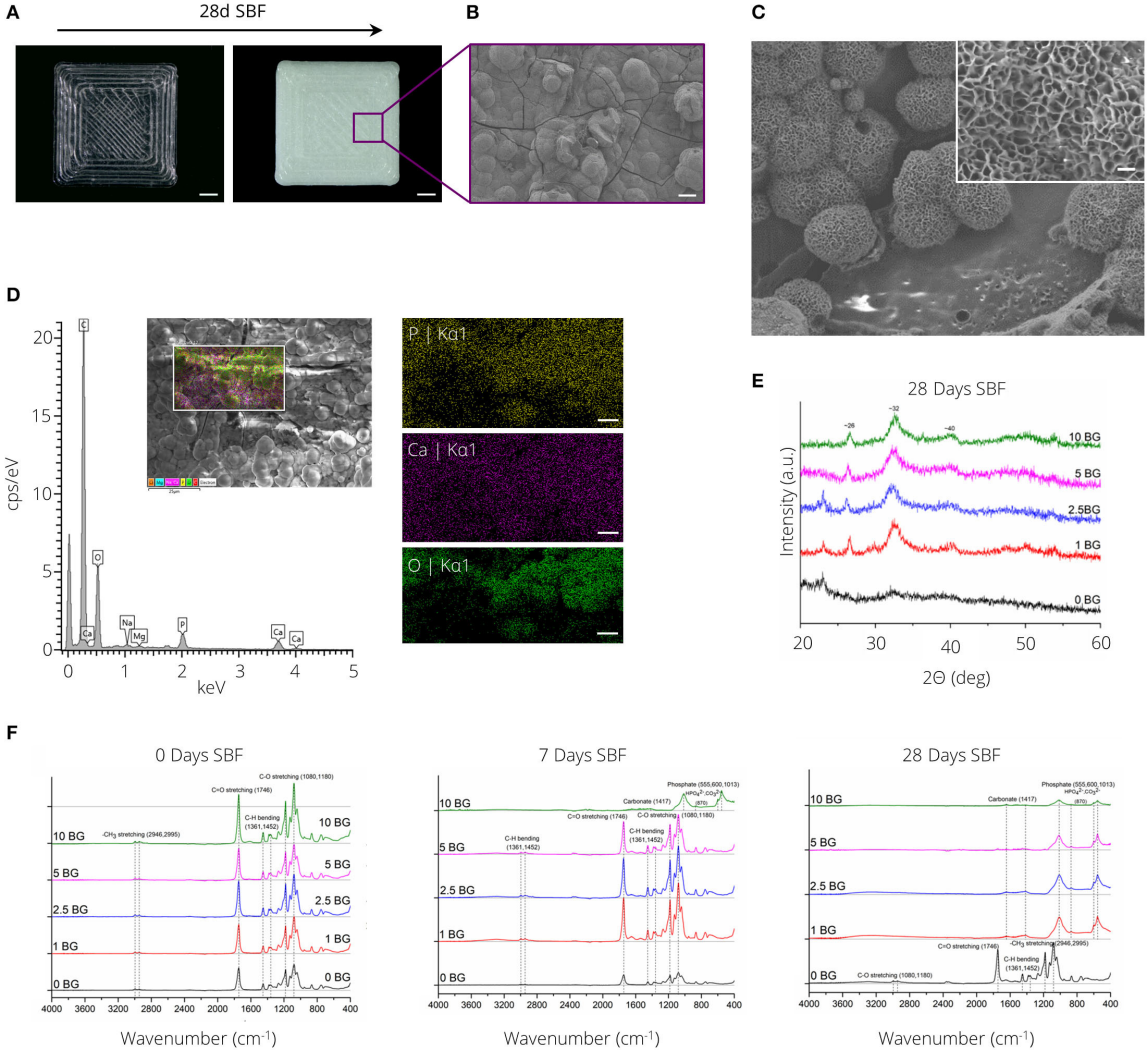
Bioactivity assessment of PLA-BG materials. **(A)** Light microscopy images of 3D printed PLA-1% BG squares before (left) and after (right) incubation in simulated body fluid for 28 days. The precipitation of a white layer is apparent on the material surface. Scale bar = 2 mm. **(B)** SEM micrograph showing the formation of a solid layer with cauliflower-like structures present, indicating the formation of a ceramic hydroxy-carbonated apatite (HCA)-like layer. Scale bar: 20 μm. **(C)** SEM image of cauliflower like structure on PLA-BG after 14 days of incubation in SBF. Scale bar: 250 nm. **(D)** Energy dispersive x-ray spectrum of PLA-10% BG scaffolds incubated for 14 days in SBF, indicating the formation of phosphorus, calcium and oxygen species on the formed layer. Scale bar: 5 μm. **(E)** X-ray diffraction spectrum of the material surfaces of PLA-BG incubated for 28 days in SBF indicating the formation of crystalline species on the surface. **(F)** Fourier-transformed infrared spectroscopy analysis of PLA-0, 1, 2.5, 5, and 10% (wt) BG squares after 0, 7, and 28 days of incubation in SBF. The formation of new peaks indicative for carbonates and phosphates is shown over SBF incubation time.

Figure 5A depicts the formation of a white layer on the surface of PLA-1% BG rectangular plates after 28 days of incubation in SBF. SEM micrographs (Figures 5B,C) in combination with SEM-EDX analysis (Figure 5D) confirmed the formation of a calcium-phosphate layer with cauliflower-like structures visible after 14 days of incubation in SBF (Figure 5C). X-ray diffraction analysis indicated the formation of a crystalline layer for all PLA compositions incorporating BG after 28 days of SBF incubation (Figure 5E). Diffraction peaks at ~26, 32, and 40° 2Θ were observed after incubation in SBF. Notably, diffraction peaks indicating layer crystallinity were observed after 14 days of SBF incubation for the highest BG incorporating (10% (wt)) PLA-BG composition (Supplementary Figure 4). FTIR absorbance spectra of PLA-BG plates after 0, 7, and 28 days of incubation in SBF (Figure 5F) showed the formation of absorbance peaks at ~1,013, 600, and 555 cm^−1^, initially for PLA-10% BG after 7 days of incubation in SBF, eventually occurring for all PLA-BG compositions after 28 days of incubation in SBF (Figure 5F).

### Cytocompatibility of PLA-BG Scaffolds

**Figure 6:**
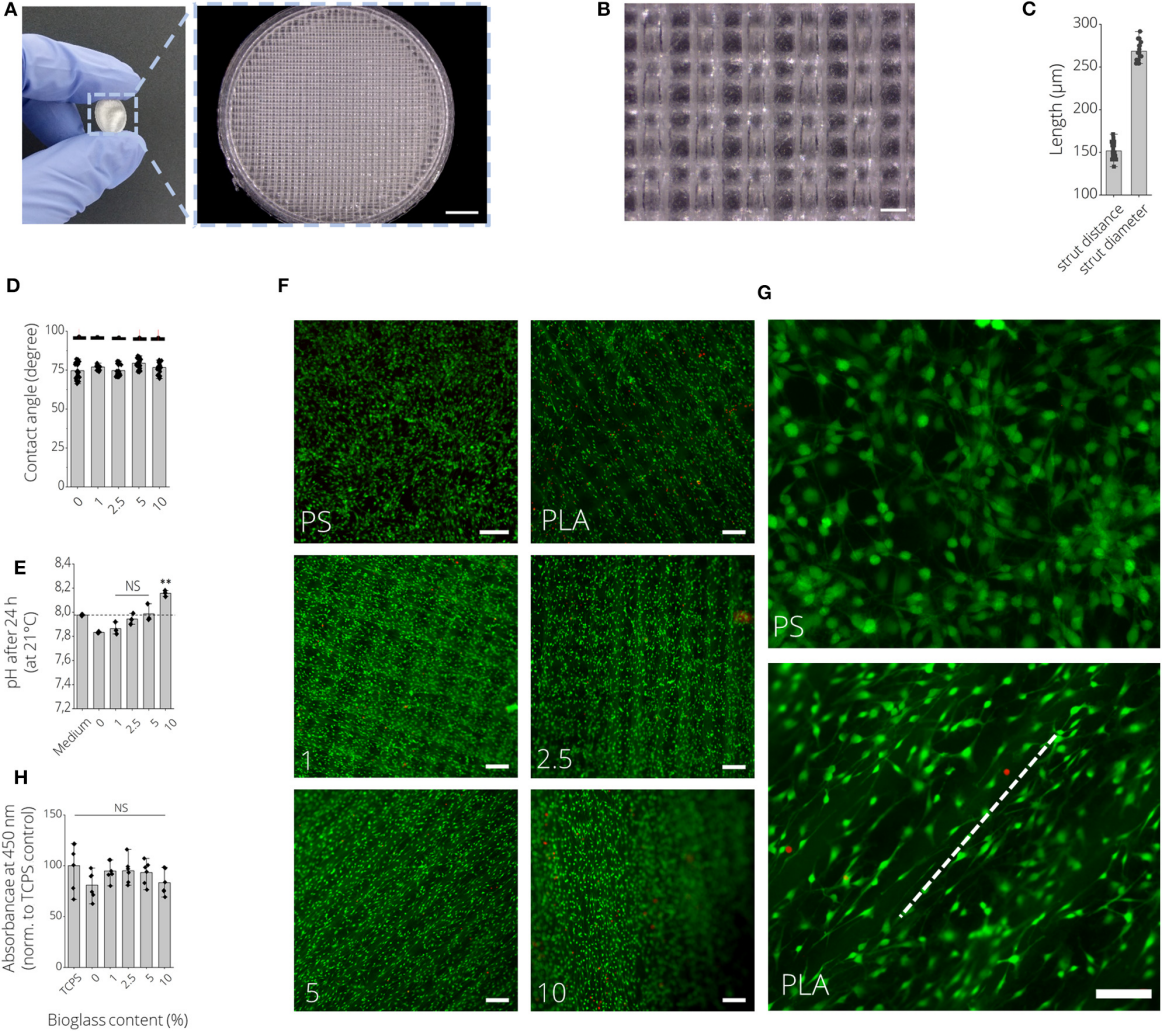
2D *in vitro* cytocompatibility assessment of FDM 3D printed PLA-BG disks. **(A)** Macroscopic image of a 3D printed PLA-BG disk used for initial cytocompatibility assessment. Light microscopy image illustrates the patterned surface of the FDM 3D printed PLA-BG disk. Scale bar: 2 mm. **(B)** Representative light microscopy image of the grit-like strut-by-strut surface structure of the cell-culture disk. Scale bar: 200 μm. **(C)** Corresponding quantification of distance between two struts and strut diameter. **(D)** Water contact angle as a measure of wettability of PLA-BG disks (*n* = 6). Data presented as mean ± SD. **(E)** Development of pH value of cell culture medium incubated with 3D printed PLA-BG disk of 0, 1, 2.5, 5, and 10% (wt) BG content over 24 h at 37°C, measured at 21°C. No significant increase in pH (NS) except for 10% (wt) BG-laden PLA disks was observed (*n* = 6). Data presented as mean ± SD. ^**^p < 0.01 compared to untreated cell culture medium by one-way ANOVA. **(F)** Fluorescence microscopy images of Calcein AM (green) and propidium iodide (red) stained MC3T3E1 cells after 24 h of incubation on PLA-BG disk and polystyrene reference substrates depicting LIVE/DEAD cells, respectively. Scale bars: 200 μm. **(G)** Cell orientation was present on PLA-BG disks (dashed white line) in comparison to PS cell culture well plates. Scale bar: 200 μm (both images). **(H)** Corresponding indirect viability WST-8 assay data with no significant (NS) difference in viability detected among the substrates.

**Figure 7:**
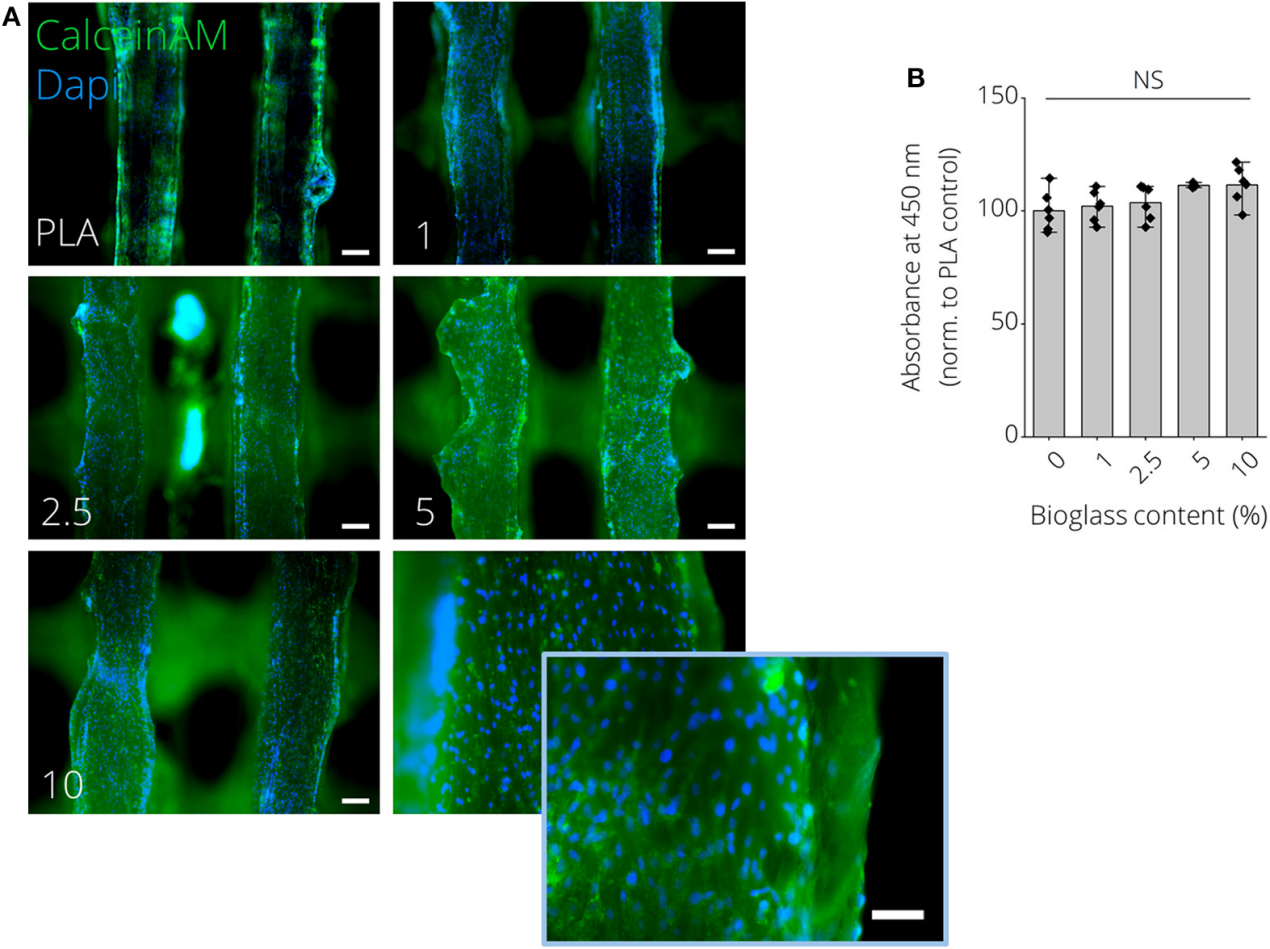
MC3T3E1 pre-osteoblast cells cultured on 3D printed PLA-BG substrates for 24 h. **(A)** Fluorescence microscopy images of Calcein AM (green)/DAPI (blue) stained MC3T3E1 on 3D printed PLA-BG scaffolds with 0, 1, 2.5, 5, and 10% BG, respectively. Scale bars: 200 μm, 100 μm (insert, bottom right). **(B)** Indirect cell viability WST-8 test of MC3T3E1 cells on different substrates (*n* = 6), with no significant difference (NS) detected in WST conversion among the different groups.

Figures 6A,B show macroscopic images of 3D printed PLA-BG disks exhibiting a patterned surface for initial cytocompatibility assessment. Cell culture disks with increasing BG content showed similar increase in turbidity, analog to the observation made for the filaments (Supplementary Figure 5). The high quality of the produced scaffolds is to be expected when using additive manufacturing with strut distances of about 150 μm and strut diameter of 250 μm (Figure 6C). The hydrophobicity (water contact angle, Figure 6D) of the PLA surfaces did not change by adding BG, while only the pH value increased with the amount of BG over 24 h at 37°C (Figure 6E). In fact, the pH increase was dependent on BG content and changed over time, suggesting BG release (Supplementary Figure 6). The initial *in vitro* cytocompatibility studies of the different 2D surfaces performed via WST-8 assay showed no significant difference in viability with an increase of BG content (Figure 6H). Fluorescence microscopy images with Calcein AM (green) and propidium iodide (red) stainings show that the cells can be guided by the structures (Figures 6F,G). MC3T3-E1 cells expressed long, elongated and fibroblastic morphology after 24 h of incubation with PLA, which can be seen in Figure 6G. Fluorescence microscopy images of Calcein AM/DAPI (blue) stained MC3T3E1 cells on 3D printed PLA-BG scaffolds with 0, 1, 2.5, 5, and 10% BG are shown in Figure 7. Translating the results from 2D to 3D reveals no negative change in cell behavior, the MC3T3-E1 cells can grow well on all scaffold surfaces and the viability (indirect assay) is not dependent on the degree of filling of the polymer with BG (Figure 7).

### Gene Expression of Human ASCs on FDM Printed PLA-BG Scaffolds

**Figure 8:**
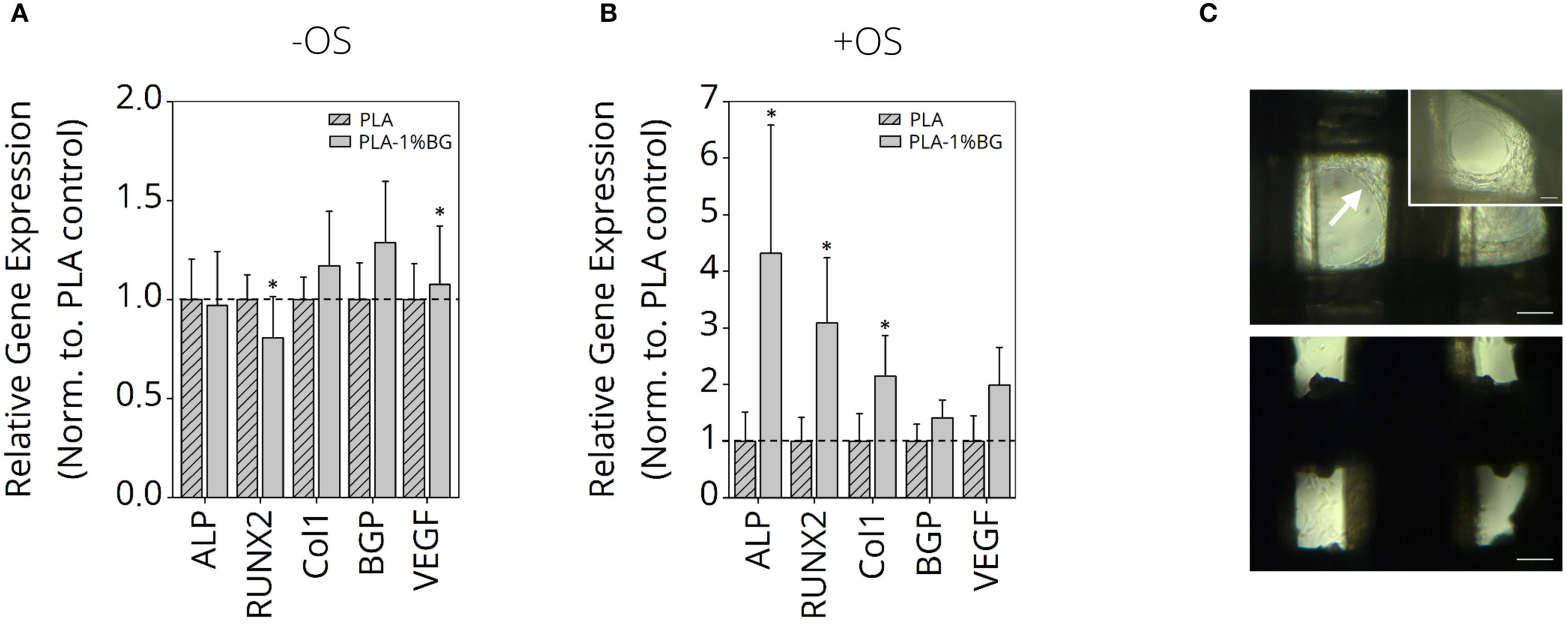
Gene expression study of human ASC on PLA-1% BG scaffolds. Relative gene expression of Col1, VEGF, BGP, RUNX2, and ALP in human ASC cultured for 35 days on 3D printed 1% BG containing PLA scaffolds normalized to PLA scaffolds in **(A)** nonosteogenic and **(B)** osteogenic differentiation medium. Data is reported as mean ± s.e.m (*n* = 4). ANOVA with *post-hoc* Tukey -HSD test; ^*^p ≤ 0.05. **(C)** Light microscopy images of ASC after 35 days cultured in non-osteogenic (top) and osteogenic (bottom) medium. The white arrow indicates higher cell growth observed inside scaffold pores for ASC cultured in nonosteogenic medium in comparison to +OS scaffolds, indicative for higher proliferation on –OS scaffolds. Scale bars: 200 μm, 100 μm (insert, top right).

Since the most promising results regarding mechanical strength and printability were gained for PLA-1% BG scaffolds, osteogenic cell differentiation with and without osteo-induction stimulants was performed (Figure 8). The relative expression of ALP, RUNX2 as osteoblast markers, Col1, Osteocalcein as ECM expression markers, as well as VEGF as an angiogenesis marker in human ASC, cultured for 35 days, was detected. While it was not possible to increase the osteoblastic differentiation in the pure PLA samples in comparison to the BG containing samples, an increased expression of the ECM markers was shown (Figure 8A). BG also induced a significantly higher VEGF RNA-value. When osteoinduction stimulants were added, marked osteoblastic differentiation with increased ECM was detected on the BG-containing samples compared to the pure PLA scaffolds (Figure 8B). Cell growth inside the scaffolds after 35 days cultured in non-osteogenic (top) and osteogenic (bottom) medium is shown in Figure 8C. The white arrow indicates higher cell growth observed inside scaffold pores for human ASC cultured in non-osteogenic medium in comparison to +OS scaffolds, indicative of a higher proliferation on—OS scaffolds.

## Discussion

In this study, we demonstrated the fabrication of PLA-BG 3D printing filaments for FDM, followed by presenting the scaffold fabrication using such composite filaments, and the characterization as well as *in vitro* assessment of the new materials. Wu et al. (2020) recently reported a feasibility study on producing PLA-HA scaffolds by FDM. Estrada et al. (2017) had previously shown the fabrication of PLA-BG by FDM, however the bioactivity of scaffolds was the main focus of the study (Estrada et al., 2017). In the present work, we demonstrate the characterization and screening of filaments with different BG contents, giving an insight into the production process and printability, the fabrication of open porous PLA-BG scaffolds from such filaments, and the control over scaffold porosity via CAD. In addition, We characterized the scaffold bioactivity, mechanical properties, and investigated the cytocompatibility by cell biology and gene expression studies. Gene expression results of human adipose-derived stem cells revealed the osteoinductive properties of FDM printed PLA-1% BG in comparison to pristine PLA. Our work provides cytocompatible and osteoinductive PLA-BG filaments that can be used in FDM to develop bioactive scaffolds for bone TE. Furthermore, FDM allows the high throughput printing of scaffolds (Supplementary Figure 8) by achieving a storable intermediate filament material which is fed in a FDM printer, which may be advantageous in comparison to solvent based approaches to fabricate PLA-BG scaffolds. The PLA-BG filaments in this study showed brittle fracture and a decrease of filament toughness and tensile strength with increasing BG content (Figure 1F). Those findings suggest the presence of a non-optimal interface bonding between BG particles and the bulk PLA. SEM images of filament cross sections (Figure 1C) reveal dark areas around the BG particles inside the PLA matrix, which indicates no strong bonding between the PLA bulk and the BG particles. This is in accordance with the hypothesis of improper interface adhesion. As a result, regardless of increasing BG content, strengthening of the PLA-BG scaffolds was not observed (Figure 4C), as it would be expected from composite theory of ceramic-laden polymer scaffolds with optimal interface bonding (Gerhard and Boccaccini, 2010). Instead, a decrease in stiffness and compressive strength was observed with increasing BG loading [≥2.5% (wt)] (Figure 4C). Drummer et al. (2012) assessed PLA-βTCP FDM printed tensile specimens, showing no notable increase in elastic modulus with increasing βTCP content. Contrarily, a tendency of decreased stress at break when increasing βTCP filler content was observed, comparing specimens processed at the same temperature (Drummer et al., 2012). The group used βTCP particles with a diameter of 5.0 ± 1.0 μm (Drummer et al., 2012), similar to the d_50_ (4.0 ± 1.0 μm) of 45S5 BG particles in the present study. We observed a similarly decreased tensile stress at break when increasing the filler content. It has been reported that the combination of hydrophobic bulk polymer and hydrophilic fillers leads to improper interface bonding, as observed in our study (Goda et al., 2013). Further work could focus on different surface modifications like particle roughness, size, and chemistry as well as bulk polymer chemistry to achieve an increased polymer/filler interface binding (Boccaccini et al., 2002, 2010; Goda et al., 2013). The properties of interface bonding might be assessed via AFM to get further insight of successful interface engineering (Goda et al., 2013). Barbeck et al. (2017) and Serra et al. (2013) recorded higher compressive strength of printed samples made from PLA/PEG/calcium-phosphate glasses via direct, solvent based printing when adding glass particles. Serra et al. reported compressive strength values of 9.11 ± 1.19 MPa (Serra et al., 2013) for PLA-5%PEG scaffolds, a value much higher than the one measured on the pure PLA scaffolds in the present study. It has to be noted that the manufacturing methods used in the previous studies (Serra et al., 2013; Barbeck et al., 2017) were direct printing processes, not having an intermediate step of FDM filament production. Second, glass filler contents of up to 50% were used, five times higher than the highest concentration of BG assessed in the current study. The differences in mechanical performance in comparison to our study could be caused by the different pore sizes and scaffold designs, as well as differences in the PLA material initially used. In combination, PEG could cause improved particle-to-bulk bonding due to its higher hydrophilicity [water content angle ~44° (Pan et al., 2015)] in comparison to PLA (~75°, Figure 6D), which could allow better interface adhesion to BG. Eqtesadi et al. infiltrated 3D printed BG scaffolds with PLA or PCL, which improved the toughness and strength of the scaffolds (Eqtesadi et al., 2014, 2016a,b). Serra et al. showed the reduction of hydrophobicity by PEG addition to PLA (Serra et al., 2013). The pore sizes in Serra et al.'s study were much smaller (375 ± 25 μm between struts) in comparison to the pore size in our work. Alongside with the different scaffold design, the change in porosity could lead to a better stress distribution in comparison to the scaffolds fabricated in our study. Drummer et al. (2012) highlighted the influence of specimen size used for mechanical characterization of FDM parts, observing that larger samples resulted in higher stiffness of the assessed material due to (i) the ability to print more homogenous specimens, being less susceptible to incorporate structural inhomogeneities and defects, and (ii) a reduced influence of the specimen surface roughness on the tensile testing in comparison to smaller specimens (Drummer et al., 2012). As a result, a combination of manufacturing related defects and potential improper binding at the BG-PLA interface might have led to the decrease in stiffness and strength of the present scaffolds, leaving room for improvement. It was intentional by the authors to choose specimens for tensile and compression testing similar to scaffolds used for *in vitro* characterization. However, a comparison of different blends of polymer and BG particles utilizing ISO tensile specimens may be a valid approach to assess polymer-filler material interaction, as demonstrated by Drummer et al. (2012). Strut diameters of around 625 μm were achieved with a D = 400 μm nozzle. We found that the first layer deposited on the glass plate tended to have a higher strut diameter (d = 635 μm) due to PLA wetting on the glass. This effect is not present when the polymer is deposited on existing struts (Figure 6C, cell culture disk, strut diameter ~275 μm). Strut diameters could be tuned by using different printing nozzles, e.g., D = 200 μm. The highest resolution of pure PLA and PLA-BG composites were strut distances of 163 ± 27 μm (Ultimaker PLA control: 108 ± 25 μm). Both materials feature similar SD (~25 μm), which can be attributed to the FDM printer. Thus, PLA and PLA-BG composites showed no statistically significant difference in printability. Pores of ~165 μm were achieved by Barbeck et al. using PLA-bioactive glass and PEG (Barbeck et al., 2017). The resolution achieved here, with the advantage of using a solvent-free approach, is comparable to PLA-BG structures obtained by direct printing reported in literature (Barbeck et al., 2017). We observed increasing SD of strut diameter and pore size with increasing BG content. This can be an indicator of a loss in printing accuracy, producing lumps and defects. One reason for this behavior can be the higher variation in filament diameter during production (Figure 1D), which may lead to defects during scaffold manufacturing in FDM. Serra et al. (2013) demonstrated direct printing of PLA combined with PEG and 50 wt% BG particles (44.5P_2_O_5_-44.5Ca_2_O-6Na_2_O-5TiO_2_ mol, d <40 μm, G5; Serra et al., 2013), independent of any filament quality. However, such direct printing approaches may require prior adjustment of polymer viscosity using solvents or plasticizers to allow 3D printing (Serra et al., 2013; Barbeck et al., 2017). Diomede et al. (2018) produced PLA scaffolds using filaments by FDM. They reported a pore size deviation of 24.1% (Diomede et al., 2018), comparable to pore size deviation of 0 and 1% BG-PLA scaffolds in our study. The study by Estrada et al. (2017), which is the closest to our work, cannot be compared regarding printability, as no similar data was reported. FTIR analyses at day 0 (Figure 5F, left) for PLA-BG depict absorbance peaks at 1,746 cm^−1^ [v(C=O)], 1,361 cm^−1^ [v(CH-CH3)], 1,452 cm^−1^ [v(CH3)], and 1,080, 1,180 cm^−1^ [v(C-O-C)], characteristic for PLA (Yuniarto et al., 2016). No characteristic peaks of BG were present. However, SEM images showing BG incorporated in PLA and positive bioactivity results indicate that BG particles are incorporated and surrounded by the bulk PLA matrix, which were not detectable by FTIR surface analysis. PLA-10% BG was the only composition showing silica peaks in EDX indicating BG on its surface. The result supports the hypothesis that BG was mostly surrounded by the PLA polymer when embedded in the polymer matrix by processing through filament making/3D printing, leaving BG particles undetectable for EDX surface analysis. However, for the highest BG composition (PLA-10% BG), a sufficient amount of BG particles was added to the matrix so that it was possible to detect BG by EDX. X-ray diffraction peaks at ~26, 32, and 40° 2Θ after incubation of PLA-BG in SBF for 28 days suggest (002), (211), and (310) lattice diffraction of hydroxyapatite (HAP) (Takemoto et al., 2004; Meena et al., 2012; Shahabi et al., 2014). FTIR analysis shows stretching vibrations at 1,417 cm^−1^, indicative of carbonate, and phosphate peaks [v(PO), v(CO)] at 555, 600, and 1,013 cm^−1^(Rehman and Bonfield, 1997), suggesting a calcium-phosphate surface layer coverage. Figure 5C showing PLA-BG scaffolds after 14 days of SBF incubation (SEM images) indicates the presence of cauliflower like structures, while Figure 5B after 28 days in SBF depicts the growth of the initial cauliflower like structure to a dense HAP layer. Summarizing, XRD, FTIR, SEM, and EDX analyses indicate the formation of hydroxy-carbonated apatite, confirming scaffold bioactivity even for 1% BG content PLA. Hence, the filaments in this study were confirmed to be bioactive. The formation of a hydroxyapatite-like layer has been shown to be crucial for successful implant-bone bonding (Boccaccini et al., 2010). Estrada et al. (2017) demonstrated bioactivity of PLA-BG composites after 7 days of incubation in SBF (crystallinity at ~20° 2Θ) (Estrada et al., 2017). We show the evolution of the calcium-phosphate layer on PLA-BG scaffolds toward higher detectable crystallinity at time points exceeding 7 days, maturating from day 14 (Figure 5C; Supplementary Figure 4) to day 28 of SBF incubation (Figures 5B,E). The results found by Estrada et al. (2017) are similar to the present XRD analysis after 14 days of incubation (Supplementary Figure 4). The wide peak forming at approx. 20° 2Θ is possibly due to semi-crystalline PLA with a slight additional peak at ~23° 2Θ (Chieng et al., 2014; Nanaki et al., 2018). PLA-10% BG shows only a main peak of hydroxyapatite at ~32° 2Θ after 14 days of incubation in SBF, suggesting accelerated bioactivity with higher BG content. The encapsulation of BG particles in the bulk PLA allows to reduce BG ion release. Therefore, the release of BG dissolution products could be controlled through BG filler content (Supplementary Figure 6), indicated by pH changes monitored over time. The ion release from, and bioactivity of the scaffolds can be controlled through the PLA resorption properties and BG filler content (Supplementary Figure 7) (Boccaccini and Maquet, 2003). *In vitro* cytocompatibility studies of PLA-BG composites in 2D showed cytocompatible surfaces independent of BG content. This result confirms that PLA-BG composite scaffolds made from extruded filaments are not cytotoxic. Kim et al. (2012) have shown that MSCs on PLA-BG composite exhibit higher cell viability after 3 days compared to pure PLA. Regarding structure compatibility, the fabricated strut-by-strut 3D-structures are well- known in TE and have already proven their potential on bone tissue scaffolds (Hollister et al., 2002; Detsch et al., 2008; Rottensteiner et al., 2014). We confirm that by 3D printing μm-range grooved patterns (150 μm) an alignment of cells can be triggered (Figure 6G), as shown for groove widths of ~842 μm (Blasiak et al., 2019). As a result, the printed PLA-BG plates could be used as platforms for cell guidance. PLA-BG scaffolds developed in this work exhibited hydrophobic surfaces independently of BG content (Figure 6D), which is the result of the intrinsic hydrophobicity of PLA caused by the presence of non-polar methyl groups (Cohn and Younes, 1988; Yang et al., 2002; Kim et al., 2006). This behavior corresponds to the fact that BG particles were incorporated in the PLA bulk and not present on the scaffold surface. PLA surface chemistry can be tailored by surface modifications or by adding hydrophilic polymers. Serra et al. (2013) showed an increase in the wettability of G5 BG containing scaffolds in contrast to our study. This difference could be attributed to the direct printing used, which might not lead to encapsulation of the BG particles in the bulk PLA compared to the FDM filaments fabricated here. Gene expression studies revealed that BG induces a higher expression of collagen and osteocalcin by human ASCs compared to pure PLA scaffolds (Figure 8A). Together, these two markers confirmed the osteogenic effectiveness of BG. The higher proliferation of non-stimulated cells is shown in Figure 8C, whereas the observed overgrowth of the squared pores is in correspondence with Rüdrich et al. (2019). They showed that scaffold pore design is of high importance for cell sensing during the initial step of cell adhesion and proliferation (Rüdrich et al., 2019). The use of +OS increased the expression of ALP and RUNX2 in cells grown on PLA-BG scaffolds, which are the characteristic markers for osteoblasts, thus confirming the potential of the PLA-BG scaffolds for bone tissue engineering.

## Conclusions

We have shown the successful fabrication of PLA-BG composite filaments for the manufacturing of 3D scaffolds by fused deposition modeling. The filaments containing BG particles of size 4.0 ± 1.0 μm (d_50_) exhibited bioactivity. It was possible to predict and control porosity and scaffold shape for PLA-1% BG filaments with similar accuracy to the commercially available PLA FDM standard. The developed PLA-BG scaffolds triggered increased osteogenic differentiation of adipose derived human stem cells *in vitro*. By this approach, a high throughput, solvent free manufacturing route of PLA-BG composite scaffolds was demonstrated, which provides a versatile and potentially patient specific biomaterial platform for bone tissue engineering.

## Data Availability Statement

All 3D printing files generated for this study are included in the article/Supplementary Material.

## Author Contributions

TD, RD, and AB designed the experiments. TD and NF conducted main experiments and analyzed the data. TD, RD, NF, and AB wrote the manuscript. TD performed manuscript formatting and data visualization. CP and HS conducted and processed μCT experiments. TD and AG conducted gene expression experiments and analysis. TD, CP, HS, RD, and AB contributed to data interpretation and commented on the manuscript. AB supervised the overall project. All authors listed, contributed directly, and substantially and intellectually to the work. This work was approved by all authors for publication.

## Conflict of Interest

The authors declare that the research was conducted in the absence of any commercial or financial relationships that could be construed as a potential conflict of interest.

## Supplementary Material

Supplementary Video 1 μCT of PLA-1%BG Scaffold.

Supplementary Video 2 μCT slicing of PLA-1%BG Scaffold.

Supplementary Video 3 Compression Test of PLA-1% BG Scaffold.
