# Supplementary material for: Polymer-Bioactive Glass Composite Filaments for 3D Scaffold Manufacturing by Fused Deposition Modeling: Fabrication and Characterization
Source: Front Bioeng Biotechnol. 2020 Jun 24;8:552. doi: 10.3389/fbioe.2020.00552 (PMC7326953; doi:10.3389/fbioe.2020.00552)
Supplement: Supplementary file 4 [file Data_Sheet_1.pdf]

Polymer-Bioactive Glass Composite Filaments for 3D Scaffold Manufacturing by Fused Deposition Modelling: Fabrication and Characterization

Thomas Distler[1], Niklas Fournier[1], Alina Grünewald[1], Christian Polley[2], Hermann Seitz[2], Rainer Detsch[1], Aldo R. Boccaccini[1,2*]

[1]Institute of Biomaterials, Department of Materials Science and Engineering, Friedrich-Alexander-University Erlangen-Nuremberg, Erlangen, Germany

[2]Chair of Microfluidics, Falculty of Mechanical Engineering and Marine Technology, University of Rostock, Rostock, Germany

*** Correspondence:**
Prof. Dr. Aldo R. Boccaccini
aldo.boccaccini@ww.uni-erlangen.de

Supplementary Information

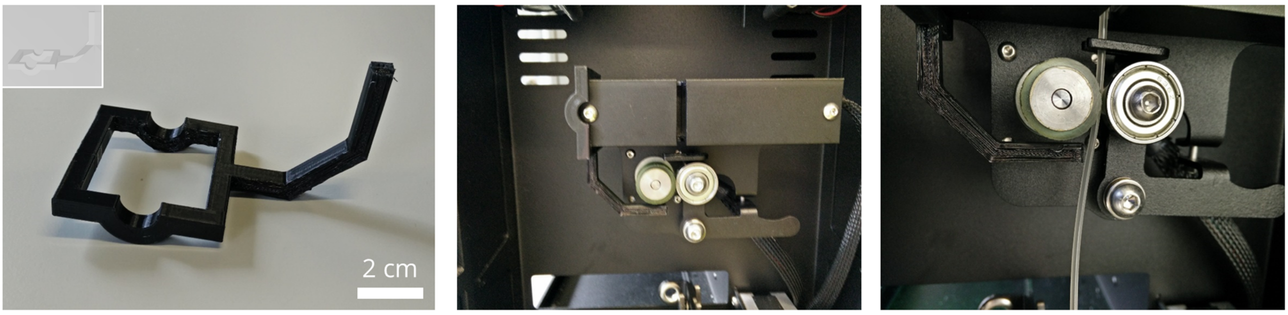

SUPPLEMENTARY FIGURE 1 | Support structure to avoid filament adhesion and coiling on the puller wheels (right image) for 3Devo NEXT Filament maker. CAD *.stl file available for download in the supporting information.

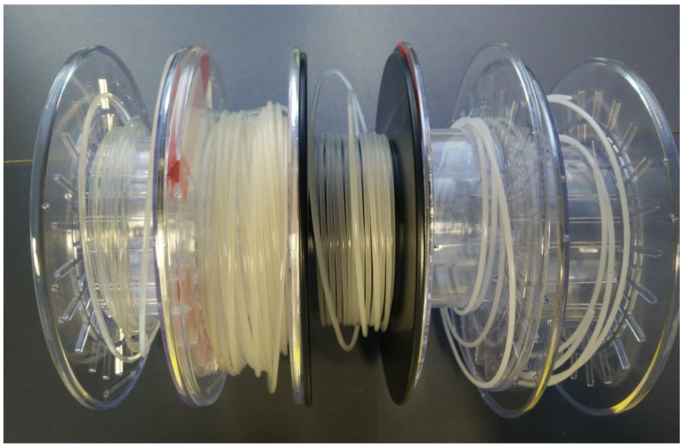

SUPPLEMENTARY FIGURE 2 | Spooled filaments of PLA-BG with different bioglass content. From left to right: PLA, PLA-1%BG, PLA-2.5%BG, PLA-5%BG, PLA-10%BG.

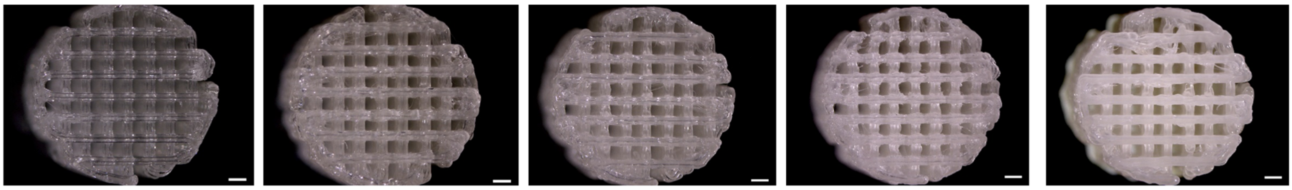

SUPPLEMENTARY FIGURE 3 | Light microscopy images of PLA-BG scaffolds with 0%, 1%, 2.5%, 5% and 10% (w/v) Bioglass (left to right). Scale bar: 1 mm.

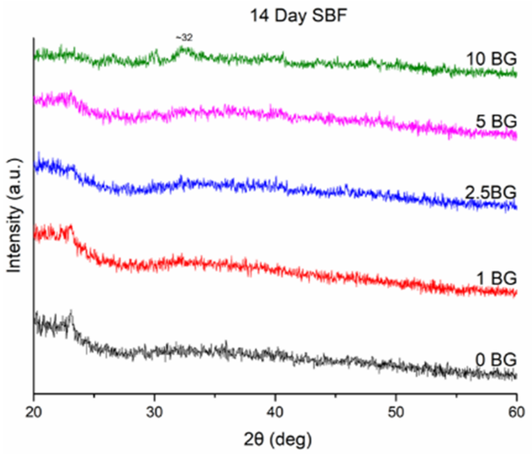

SUPPLEMENTARY FIGURE 4 | X-ray diffraction analysis (XRD) of PLA-BG squares after 14 days of incubation in SBF. Peak development at ~32° 2Θ indicating crystalinity for PLA-10% (wt)BG composition of highest BG content.

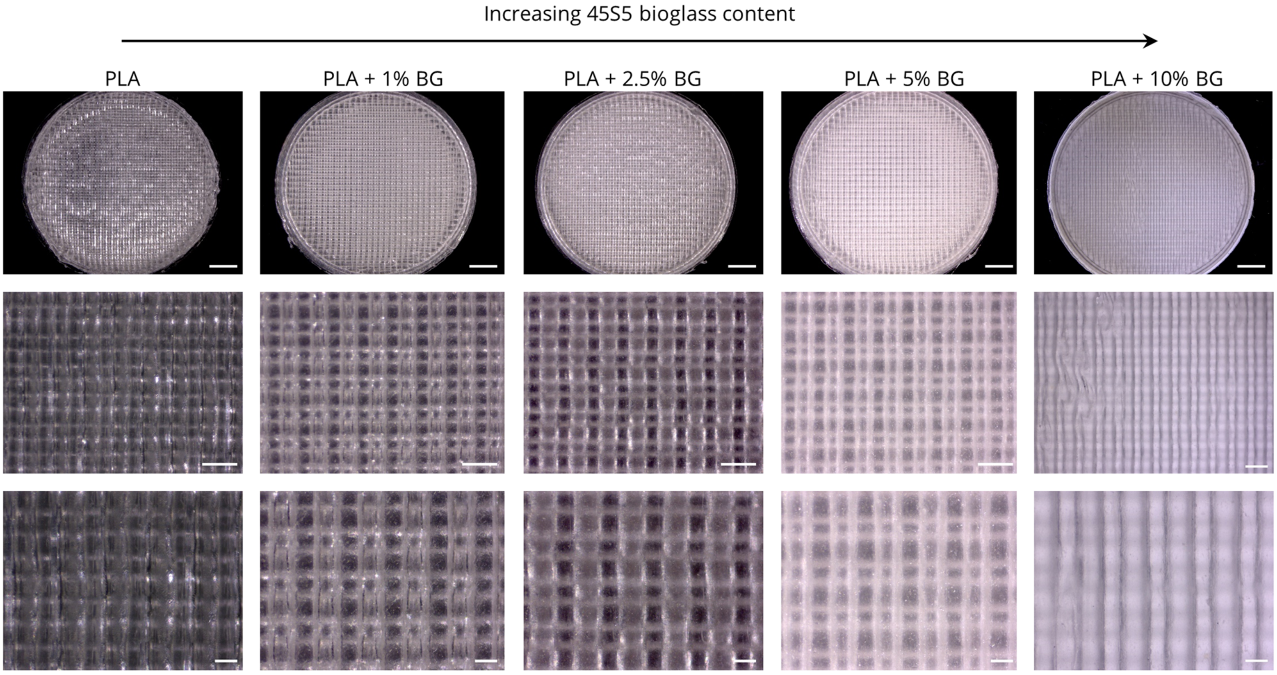

SUPPLEMENTARY FIGURE 5 | 3D printed PLA-BG discs for in-vitro biocompatibility assessment. Light microscopy images illustrating patterned surface of 3D FDM printed PLA-BG discs; scale bars: 2000 µm (top row), 500 µm (mid row), 200 µm (bottom row).

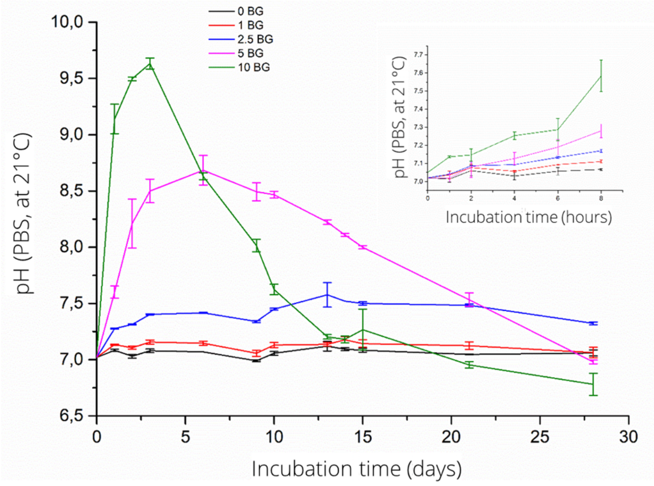

SUPPLEMENTARY FIGURE 6 | 3D printed PLA-BG discs immersed in PBS at 37°C for different time points. pH development depicted with rapid pH increase for 5% and 10% (wt) BG containing PLA associated to BG dissolution.

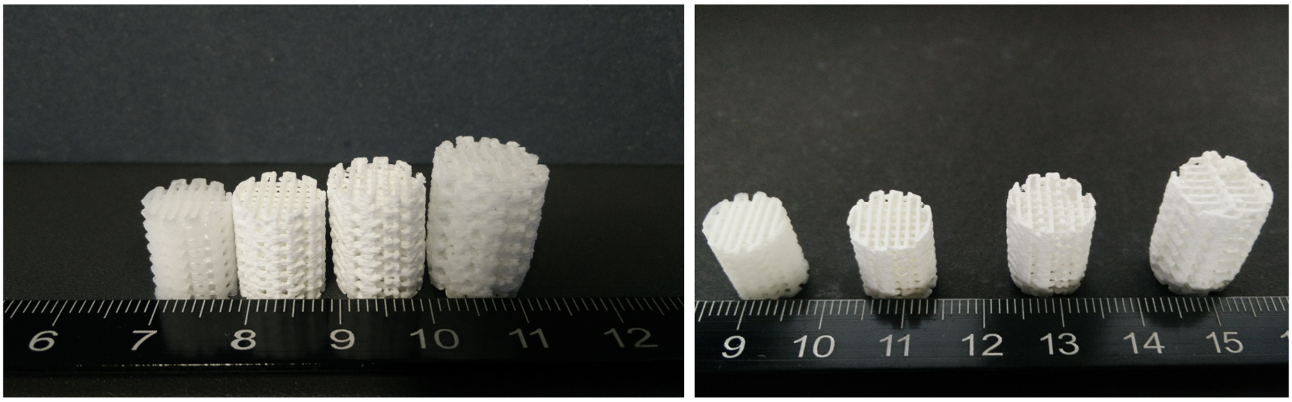

SUPPLEMENTARY FIGURE 7 | 3D printed PLA-BG scaffolds incubated in PBS for 28 days. From left to right: PLA, PLA-1%BG, PLA-2.5%BG, PLA-5%BG scaffolds. Scaffold swelling is indicated with increasing bioglass content.

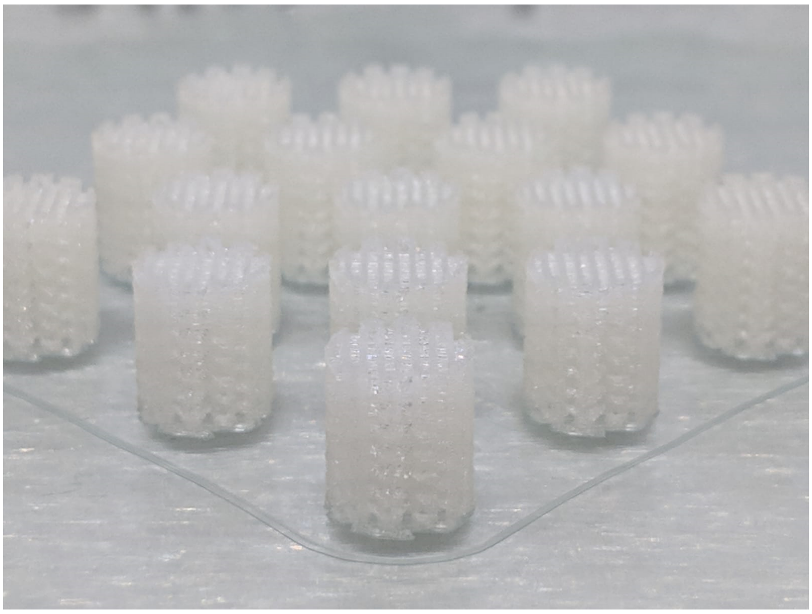

SUPPLEMENTARY FIGURE 8 | Simultaneous manufacturing of sixteen PLA-BG scaffolds in one print using the 3D printer

Supplementary TABLE S1| Printing parameter details for producing PLA-BG scaffolds.

Parameter	Value
Print core	0.25
Ahesion type	Skirt
Layer height	0.2
Layer height 0	0.1
Prime tower enable	False
Position	0
Bottom layers	5
Infill overlap	0
Infill pattern	Lines
Infill sparse density	100
Initial layer line width factor	100
Line width	0.4
Mateiral flow	100
Material print temperature	160
Build plate temperature	55
Retraction hop enabled	false
Speed layer 0	25
Speed print	30
Speed topbottom	25
Speed travel	100
Speed travel layer 0	100
Speed wall	25
Speed wall 0	25
Top layers	5
Wall line count	1
Wall thickness	0.7
Generate support	false

SUPPLEMENTARY Table S2 | PCR primers used and genes investigated in qRT-PCR analysis.

Gene	Gene name	PrimePCR Assay ID	Gen Bank No.
COL1A1	collagen type I (col I)	qHsaCED0043248	NG_007400.1
VEGFA	vascular endothelial growth factor A (vegf)	qHsaCED0043454	NG_008732.1
BGLAP	bone gamma-carboxyglutamate protein (bgp, osteocalcein)	qHsaCED0038437	NT_004487.20
RUNX2	runt-related transcription factor 2 (runx2)	qHsaCID0006726	NG_008020.1
ALPL	alkaline phosphatase (alp)	qHsaCID0010031	NC_000001.10
GAPDH	glyceraldehyde-3-phosphate dehydrogenase	qHsaCED0038674	NC_000012.11
YWHAZ	14-3-3-zeta	qHsaCID0013897	NC_000008.10
HPRT1	hypoxanthine phosphoribosyltransferase 1	qHsaCID0016375	NC_000023.10
